# Predictors of high- and low-risk drinking after group treatment for alcohol use disorder

**DOI:** 10.1192/bjo.2025.10047

**Published:** 2025-07-14

**Authors:** Kristoffer Høiland, Espen Kristian Ajo Arnevik, Lien My Diep, Tove Mathisen, Anette Søgaard Nielsen, Jens Egeland

**Affiliations:** Department of Addiction Treatment, Vestfold Hospital Trust, Tonsberg, Norway; Department of Psychology, University of Oslo, Norway; Department of Addiction Treatment, Oslo University Hospital, Oslo, Norway; Oslo Center for Biostatistics and Epidemiology, Research Support Services, Oslo University Hospital, Oslo, Norway; Blue Cross Clinic Skien, Skien, Norway; Psychiatric Research Unit, Department of Clinical Research, University of Southern Denmark, Odense, Denmark

**Keywords:** Alcohol use disorder, treatment, outcomes, patient factors, predictors

## Abstract

**Background:**

Understanding the factors influencing alcohol use disorder (AUD) treatment outcomes is essential. More knowledge about patient characteristics that predict treatment outcomes can help personalise interventions, improve treatment planning and address the needs of specific subgroups. The frequency of treatment attendance may also affect drinking outcomes after treatment. Despite research efforts, uncertainty remains about how patient factors and treatment attendance influence treatment outcomes.

**Aims:**

To examine how patient factors and treatment attendance predict high- or low-risk drinking at the end of treatment.

**Method:**

We used data (*N* = 92) from a multisite observational study of treatment-seeking individuals with AUD attending group treatment. Sociodemographic measures, alcohol and substance use measures, cognitive functioning, psychological distress, personality functioning and quality of life were screened in univariate analyses. Significant variables were entered into a binary logistic regression model.

**Results:**

Individuals with a higher percentage of treatment attendance (odds ratio 0.96 [95% CI 0.93, 0.96]) and with greater responsiblity scores on the Severity Indices of Personality Functioning (odds ratio 0.30 [95% CI 0.14, 0.64]) had a decreased likelihood of high-risk drinking at treatment end. Substance use, psychological distress and cognitive functioning were not associated with drinking levels at the end of treatment.

**Conclusion:**

A higher percentage of treatment attendance has a minor effect on drinking levels. Being more responsible, as reflected in higher scores on the responsibility domain, reduces the likelihood of high-risk drinking at the end of treatment. Clinicians are encouraged to screen and assess personality functioning when planning treatment for individuals with AUD.

Understanding how subject characteristics influence the outcomes of alcohol use disorder (AUD) treatment is crucial for clinical practice. Regardless of treatment modality, factors predictive of treatment outcome may improve personalised interventions, address the needs of specific subgroups and influence treatment planning and prognostic considerations.^1,2^ Despite numerous investigations of outcome predictors, there is still uncertainty regarding how patient characteristics and attendance in treatment influence AUD treatment outcomes.

The severity of alcohol problems or alcohol consumption pretreatment,^1,2^ a history of previous AUD treatment^3^ and level of education^3,4^ are among subject characteristics that have been suggested as potential predictors of poor outcomes following treatment. Other risk factors include physical health and comorbid medical problems,^4^ lack of social support or pro-drinking networks,^2,5^ psychological distress,^1^ certain personality traits^1,6^ and morphological brain changes and neuropsychological impairments.^4,7,8^ Correspondingly, a literature review examining relapse factors for persons with AUD found that AUD severity, psychiatric comorbidity, comorbid drug use and health and social factors were risk factors for recurrence of drinking.^9^ The authors also determined that social networks, self-efficacy and meaning in life were protective factors. However, this review included an array of study designs on AUD and relapse, with conclusions drawn from both clinical and non-clinical populations. Thus, care should be taken in extending these findings to treatment-seeking groups.

Personality disorders and maladaptive personality functioning are common in AUD,^10^ with one study estimating a 23.8% prevalence of personality disorders in a sample of individuals from the general population with AUD.^11^ However, prevalence rates vary significantly between studies, and recent research with large samples is lacking.^6^ Personality problems are related to potentially less favourable treatment outcomes.^6,12^ They should, therefore, be investigated further because there is a lack of studies examining the association between outcomes in the treatment of AUD and personality.^10^

During the past decades, altough cognitive functioning has also been suggested as a critical factor influencing outcomes in AUD treatment,^7,8^ the findings are still inconsistent. There are no clear-cut cognitive profiles of AUD, and the underlying mechanisms explaining exactly how cognitive impairments affect treatment are not fully understood (e.g. ^8^).

Furthermore, treatment attendance, such as the number of sessions attended and the related concepts of treatment retention and completion, has also been linked to better outcomes.^3,13,14^ However, the findings are inconsistent because other studies have found either no or weak associations among treatment attendance, duration of treatment and outcome.^15,16^

Complicating the matter further, there is no agreed-upon definition of what constitutes a favourable AUD treatment outcome.^17^ Some people aim for complete abstinence, others for controlled drinking or harm reduction.^18^ Thus, the term relapse has different connotations depending on the treatment goal, and literature reviews have found no uniform definition of this concept,^9^ with some arguing for abandoning the concept altogether.^17^ In addition, there has been a call for outcome definitions to move beyond the binary drinking outcomes of complete abstinence or drinking.^19^ Drinking reduction, or controlled drinking, is established as a viable goal in research.^20^ AUD treatment in Norway also considers drinking reduction an option for a number of individuals.

In the present paper, we aimed to investigate how baseline patient factors and treatment attendance predict drinking outcomes (high- or low-risk drinking) at the end of treatment. As a naturalistic study examining individuals in standard substance use disorder (SUD) treatment clinics, variables were selected based on the characteristics and factors usually examined by clinicians in routine practice. These factors are important for understanding the individual’s course through the treatment, in particular in identifying attributes that signal the risk of poor treatment outcomes and the need for personalised interventions. We also ensured that the measures used were widely available and feasible for administration by clinicians. We chose the outcome variable of low- versus high-risk drinking, because this will include individuals having either reduced or gained control over their alcohol use.

## Method

### Participants and procedures

This study used data from a multisite, longitudinal, prospective study on patient factors predicting outcomes in group treatment of AUD. One hundred and thirty-six participants were recruited from four treatment sites in Eastern Norway, two out-patient clinics and two residential AUD treatment facilities. The study was approved by the Regional Committee for Medical and Health Research Ethics South-east Norway (REK) (REKid: 125666). All participants signed an informed consent.

Participants were recruited between 2021 and 2023. Data from 92 individuals were used in the analyses for the current investigation; these were participants with data on key variables at treatment start and end. Consequently, the subjects included in this study are classified as treatment completers because they completed the planned episode and were available for assessment at the end of treatment. The difference between participants who completed and discontinued treatment was examined and has been published in a separate paper.^21^ The sample was recruited from referrals to the study sites as part of Norwegian Specialist Addiction Health Services.

All participants had AUD as their primary diagnosis, and all fulfilled the criteria for an ICD-10 diagnosis of F10.1 or F10.2, harmful use of alcohol or alcohol dependence. Ninety per cent of participants had a diagnosis of alcohol dependence or severe AUD.

We did not apply any exclusion criteria, except for (a) not being willing to participate in the baseline assessment for the study or (b) not being able to participate in the baseline assessment for the study due to cognitive, mental or somatic reasons. All patients enrolled in the study began time-limited group treatment at the treatment sites. Patients received what is regarded as standard AUD treatment in Norway, as outlined by the Norwegian Health Directorate,^22^ including psychoeducation and psychotherapeutic group treatment. Recruiting from standard treatment facilities representative of the specialist health services allowed us to capitalise on realistic treatment conditions. This increases the possibility of maximising ecological validity and translating findings into clinical practice.

Measures and background information were collected by clinical staff at the different treatment centres at the start and end of treatment.

### Measures

#### Demographic information

Demographic information was retrieved from medical records and interviews with participants. Any missing or discrepant information in medical records was sought for confirmation in the interviews.

#### Alcohol use

The Alcohol Use Disorders Identification Test (AUDIT) is a validated screening test^23^ and is currently the instrument used most widely internationally to identify hazardous or harmful drinking.^24^ There is evidence that AUDIT is a sensitive instrument in assessment of alcohol dependence severity in the clinical setting, extending its utility beyond its original use as a brief screening tool.^25^ AUDIT is also frequently used as an outcome measure in alcohol intervention trials.^26^

AUDIT comprises 10 questions yielding a score of between 0 and 40, addressing different areas: consumption, dependence and alcohol-related problems. A score of 0–7 indicates low-risk drinking or abstinence, 8–15 indicates medium-risk alcohol problems, 16–19 indicates high risk and 20–40 indicates a very high risk of alcohol problems. To measure who achieved low-risk drinking or not at the end of treatment, we constructed a binary outcome variable from the AUDIT score: LOW-RISK, abstinence or AUDIT score 0–7, and HIGH-RISK: AUDIT score 8–40. The high-risk group encompassed individuals with any problematic and harmful alcohol use following treatment; previous studies have shown that a cut-off of 8 offers good sensitivity and specificity in detecting harmful consequences of alcohol use.^27^ Participants were instructed to report their alcohol use during the treatment period. To ensure that the historical items on AUDIT did not interfere with the total score, we inspected the consumption items (items 1–3). Participants who did not report present alcohol use on these items were classified as LOW-RISK, even if their scores on the other items exceeded a total score of 7.

#### Other drug use

Comorbid drug use was measured using the Drug Use Disorders Identification Test (DUDIT), a well-validated, 11-item, self-report questionnaire for drug-related problems.^28^ Responses are scored on a scale from 0 to 4, and DUDIT yields a maximum score of 44. A total score of 0–1 indicates low risk, 2–19 increasing risk, 20–29 high risk and 30–44 very high risk.

#### Percentage of treatment attendance

Staff at the different treatment sites recorded participants’ attendance in group treatment. We calculated attendance as a percentage based on the number of sessions attended versus the number of planned sessions that participants were expected to attend. We followed this procedure as the data were collected from routine practice, and the number of treatment sessions that participants could attend varied between sites. The median number of maximum sessions possible to attend across sites was 18, the median percentage of attendance was 84.4% and the individual percentage of attendance varied between 32 and 100.

#### Neuropsychological functioning

Participants’ neuropsychological functioning was assessed with subtests from the Wechsler Adult Intelligence Scale (WAIS-IV)^29^ and the Delis–Kaplan Executive Function System (D-KEFS).^30^ WAIS-IV is a standardised test of general cognitive ability with ten core and five supplemental subtests. D-KEFS is a comprehensive test battery designed to assess a wide range of verbal and non-verbal executive functions; it consists of nine subtests.

A measure of general intellectual functioning, the General Ability Index (GAI), and three cognitive domain scores describing key cognitive abilities for everyday functioning, were derived from these tests. GAI is presented as IQ scores, with a mean of 100 and standard deviation of 15. All other test scores from WAIS-IV and D-KEFS were transformed into scaled scores, with a mean of 10 and standard deviation of 3.

GAI was estimated from four subtests derived from WAIS-IV: two verbal comprehension subtests (similarities and information) and two perceptual reasoning subtests (block design and visual puzzles). Working memory was assessed using digit span, conditions backward and sequencing.

Mental processing speed was assessed using the colour word test (CWT), conditions 1 and 2, and the trail-making test (TMT), conditions 2 and 3 from the D-KEFS. Last, executive functioning was examined using CWT conditions 3 and 4 and TMT condition 4.

#### Psychological distress

Psychological symptoms were measured using Symptom Checklist 90 (SCL-90-R),^31^ a self-report inventory of 90 questions scored on a five-point scale (0–4). Psychological distress was described using the General Symptom Index (GSI). SCL-90-R is a measure widely used for psychological distress, also in AUD and SUD populations.^32^

#### Quality of life

We used the World Health Organization Quality of Life Scale (WHOQOL-BREF)^33^ to measure quality of life. This consists of 26 items scored on a 1- to 5-point Likert scale and measures quality of life in four domains: physical health, mental health, social and environmental. Domain scores are scaled in a positive direction: higher scores denote higher quality of life.

#### Personality functioning

The Severity Indices of Personality Functioning (SIPP-118) measures core elements of maladaptive personality functioning.^34^ SIPP-118 contains 118 items covering 16 facets, constituting five higher-order domains: self-control, identity integration, responsibility, relational functioning and social concordance; response categories range from 1 to 4 (entirely disagree to fully agree), with higher total scores indicating more adaptive functioning.

### Statistical analyses

STATA/MP (v.18.0 for Windows) was used for statistical analyses. We inspected assumptions of normality using histograms and QQ plots. Chi-square and *t*-tests were performed to check for gender and age differences in the categorical outcome variable.

To examine the magnitude of change in alcohol use before and after treatment, we computed a change score for participants’ AUDIT between baseline and treatment end.

First, we screened for baseline variables significantly associated with high- or low-risk drinking. We also examined whether the percentage of treatment attendance was associated with the binary drinking outcome. We conducted univariate analyses using two-tailed *t*-tests, Mann–Whitney *U*-tests and chi-square tests, depending on the distribution. The *P*-level was set to 0.05. Due to the large number of bivariate comparisons, we applied a Bonferroni correction to the analyses. Variables that remained significant were included in the binary logistic regression prediction model. Collinearity was examined using Spearman correlations and variance inflation factors (VIFs). The Hosmer–Lemeshow test was used to examine the model’s goodness of fit to the data. Marginal probabilities were estimated with the Delta method.

## Results

### Univariate analyses

Before analysing predictors of treatment related to alcohol reduction, we needed to assess whether a reduction in alcohol consumption had occurred. The group reduced their drinking while in treatment, and mean change scores between pretreatment and treatment end were 9.66 (s.d. = 11.63) points on AUDIT. This corresponds to Cohen’s *d* of 0.83, suggesting a large effect size.

There were no significant age or gender differences in the outcome measure at the end of treatment. Table 1 presents the demographic characteristics of the sample at baseline. Around two-thirds of the sample was male, and mean age was 50 years.

**Table 1:** Demographic characteristics (*N* = 92)

Demographic characteristics	Total
Male/female, *n* (%)	61 (66.3)/31 (33.7)
Age, mean (s.d.)	50.3 (11.2)
Education (years), mean (s.d.)	13.1 (2.5)
Higher education, *n* (%)	36 (39.1)
Married/cohabiting, *n* (%)	28 (30.4)
Previous SUD treatment episodes, median (IQR)	1.0 (0–2.5)

SUD, substance use disorder; IQR, interquartile range.

Table 2 presents the results from the univariate analyses. Pretreatment drinking, other drug use, psychological distress, quality of life and neuropsychological variables pretreatment were not significantly associated with either high- or low-risk drinking at the end of treatment. The personality domains of self-control (*P* = 0.024) and social concordance (*P* = 0.021) were initially significant but not following Bonferroni correction. Only the percentage of attendance in treatment and the responsibility domain from SIPP-118 showed significant associations with outcome following Bonferroni correction, and were included in the prediction model.

**Table 2:** Associations between key variables pretreatment and outcome at treatment end (low- versus high-risk drinking)

Key variables	Total *N* (92)	Low-risk 39 (42%)	High-risk 53 (58%)	*P*-value	Missing
Demographics					
Male/female, *n*	61/31	26/13	35/18	0.950	0
Age (years), *n* (%)	50.3 (11.2)	50.0 (9.8)	50.5 (12.2)	0.820	0
Education (years), *n* (%)	13.1 (2.5)	12.8 (2.4)	13.3 (2.6)	0.391	3
Higher education, *n* (%)	36 (40.0)	15 (39.5)	21 (40.4)	0.931	2
Married, *n* (%)	28 (30.4)	14 (35.9)	14 (26.4)	0.329	0
Previous SUD treatment, median (IQR)	1.0 (0–2.5)	1.0 (0–2)	1.0 (0–3.5)	0.251	4
Substance use					
AUDIT, median (IQR)	22.0 (19.0–27.0)	21.5 (14.7–22.6)	23.0 (21.4–25.1)	0.138	3
DUDIT, median (IQR)	0 (0–12.0)	0 (1.9–8.1)	0 (4.1–10.2)	0.154	3
Percentage attendance treatment, median (IQR)	84.4 (68.4–94.9)	93.0 (82.5–92.6)	76.5 (72.8–81.7)	**0.002** ^a^	0
Cognitive functioning					
General ability index, median (IQR)	95.0 (12.8)	95.2 (13.8)	94.9 (12.2)	0.904	2
Working memory, median (IQR)	8.9 (2.2)	8.6 (2.3)	9.2 (2.1)	0.183	2
Executive functioning domain, median (IQR)	9.4 (2.4)	9.6 (2.7)	9.3 (2.2)	0.642	3
Mental speed domain, median (IQR)	9.0 (2.3)	8.8 (2.5)	9.1 (2.2)	0.574	3
Mental health and personality					
SCL-90-R (GSI), median (IQR)	0.98 (0.59)	0.93 (0.59)	1.0 (0.59)	0.623	1
Self-control, median (IQR)	5.33 (0.96)	5.61 (0.74)	5.13 (1.06)	**0.024**	9
Identity integration, median (IQR)	4.11 (0.85)	4.29(0.77)	3.98 (0.89)	0.099	9
Responsibility, median (IQR)	4.75 (0.78)	5.14 (0.65)	4.47 (0.75)	**<0.001** ^a^	9
Relational capacities, median (IQR)	4.57 (0.76)	4.76 (0.73)	4.44 (0.76)	0.056	9
Social concordance, median (IQR)	6.30 (0.69)	6.51 (0.66)	6.16 (0.69)	**0.021**	9
Quality of life					
Physical, median (IQR)	13.12 (2.73)	13.27 (2.66)	13.01 (2.80)	0.674	6
Psychological, median (IQR)	11.94 (2.69)	12.12 (2.52)	11.80 (2.84)	0.589	6
Social, median (IQR)	13.05 (2.83)	13.19 (2.93)	12.95 (2.79)	0.704	6
Environmental, median (IQR)	14.49 (2.46)	14.65 (2.70)	14.36 (2.28)	0.583	6

SUD, substance use disorder; IQR, interquartile range; AUDIT, Alcohol Use Disorders Identification Test; DUDIT, Drug Use Disorders Identification Test; SCL-90-R (GSI), Symptom Checklist 90 (General Symptom Index).

a Significant following Bonferroni correction.

All predictor variables were examined at baseline, except for the percentage of treatment attendance. Group differences are described based on two-tailed *t*-tests, means and standard deviations, or based on Mann–Whitney *U*-tests, medians and IQR for non-normal variables and chi-square tests for categorical variables.

Mental health: GSI from SCL90-R: personality domain scores from Severity Indices of Personality Functioning; quality of life: domain scores from World Health Organization Quality of Life Scale.

*P*-values <0.05 are indicated in bold.

### Multivariate analyses

The percentage of treatment attendance and the responsibility domain were entered into a binary logistic regression model.

The results are given in Table 3, and show that a 1-unit increase in treatment attendance led to a 4% decrease in the odds of high-risk drinking at treatment end. Likewise, a 1-unit increase in the responsibility domain accounted for a 70% decrease in the odds of high-risk drinking at treatment end. Marginal probabilities were estimated for key values for the predictors in the model: 25% attendance led to a predicted probability of 92% for high-risk drinking at treatment end; at 50% attendance it was 81%, at 75% attendance it dropped to 63% and at 99% attendance the predicted probability of high-risk drinking further decreased to 43%. Similarly, individuals with scores on the responsibility domain at the 25th percentile of the sample had a 73% probability of high-risk drinking at treatment end, those at the 50th percentile 56% probability, those at the 75th percentile 42% probability and those at the 99th percentile 25% probability.

**Table 3:** Odds ratios for high-risk drinking at treatment end, defined by AUDIT cut-off of 8

Predictor	Odds ratio	*P*-value	95% CI		Nagelkerke *R* ^2^
Model					0.322
Percentage attendance	0.96	**0.019**	0.93	0.99	
Responsibility	0.30	**0.002**	0.14	0.64	

AUDIT, Alcohol Use Disorders Identification Test; percentage attendance, percentage of treatment attended.

Responsibility domain from Severity Indices of Personality Functioning.

*P*-values <0.05 are indicated in bold.

The model fitted well to the data, as indicated by the Hosmer–Lemeshow test (*X* ^*2*^ [8] = 11.215, *P* = 0.190). Finally, we examined potential issues with collinearity in the model. Spearman rank-order correlations showed a weak correlation between percentage of attendance and responsibility (*r*_s_ = 0.28, *P* = 0.01), suggesting that no strong association between the two variables. A VIF of 1.05 also indicates no issues with collinearity, because VIF <5 indicates low multicollinearity.

## Discussion

In the present study, we examined potential predictors of high- and low-risk drinking at treatment end in individuals with AUD in group treatment. Our results showed that the percentage of attendance in treatment had a small but significant influence on high- or low-risk drinking after treatment. However, the personality functioning domain of responsibility was demonstrated to have the most significant impact on drinking level following treatment. Individuals who are more responsible and have the capacity to set and achieve realistic goals are more likely to achieve low-risk drinking or abstinence after completing treatment. Factors found to predict outcomes in other studies, including overall psychological distress, quality of life, the severity of drinking before treatment, other drug use and neuropsychological functioning, were not associated with high- or low-risk drinking at treatment end in the current study.

It is notable that neither general intellectual functioning nor executive functioning affected treatment outcomes in this study. This is an important finding, because it suggests that these individuals may also benefit from standard treatment for AUD, thus arguing against a common belief that they do not. However, individuals with impaired executive functioning may benefit from interventions targeted at retaining them in treatment.

Attendance in treatment has been hypothesised to be an essential factor in improving psychological distress and reducing alcohol use, because it is necessary to attend therapy to benefit from the interventions. As discussed above, research supports this idea (e.g. ^13,14^), but results are not unequivocal because some studies have failed to find a relationship between attendance and improvement. However, our findings indicate that attendance is important, at least for short-term outcomes, although the effect was not strong. Another factor, the personality domain of responsibility as measured by SIPP-118, had a much more substantial impact on short-term outcomes. This personality domain involves the individual being able to set goals and achieve these goals in line with their expectations and those of others. Furthermore, it is closely related to ‘the big five’ factor of conscientiousness.^35,36^ Thus, it is closely related to the skills necessary to attend treatment. Theoretically, treatment attendance and responsibility can be construed as closely related and dependent upon each other but, in our sample, they were only weakly correlated. Furthermore, the facets of the responsibility domain exceed the capability of attending the sessions. This also reflects the ability to set goals and remember and apply the interventions provided in therapy. As such, it is also connected to the construct of therapeutic alliance, where agreement on tasks and goals is an important factor that predicts outcomes.^37^ It probably also underlines that the individual is in a position to set a goal of low-risk drinking and abstinence and adhering to it throughout the treatment.

With a mean score on the responsibility domain of 4.47, the high-risk group’s scores are equivalent to those of individuals with one diagnosable personality disorder (mean score of 4.59).^34^ The low-risk group had higher scores and, thus, better functioning (mean score 5.14) than individuals who did not meet the criteria for personality disorder in the normative sample (mean score 4.84). Accordingly, individuals with high-risk drinking in our sample may have some degree of personality pathology, but participants were not assessed for personality disorders in the present study. However, we do know from the medical records that few individuals had a previous diagnosis of personality disorder. Whether this is due to the absence of personality pathology or lack of screening and assessment for personality pathology, we do not know.

In addition, responsibility is related to the construct of impulsivity, the ability to inhibit or withhold responses, encompassing both trait and behavioural impulsivity.^38^ There may be some degree of overlap between responsibility and impulsivity, because individuals scoring high on responsibility can control and withhold responses more effectively than those with lower scores. In contrast to responsibility, the construct of impulsivity has attracted considerable research interest and is a driving factor in the initiation of addictive behaviours,^39^ but may also be crucial for treatment outcomes.^38^ In our opinion, responsibility covers a broader set of traits and behaviours and is a viable construct to explore in future studies of individuals with AUD.

Moreover, responsibility may be an important underlying factor explaining the association between other individual factors and treatment outcomes. Findings concerning sociodemographic characteristics and outcomes are inconsistent.^1,12^ Potentially protecting factors, such as caring for children (e.g. ^40^), could be a marker of responsibility rather than a causative factor. Future studies should explore whether the ability to act responsibly mediates or moderates sociodemographic findings related to income, marriage or education level.

The clinical implications of the results are twofold: first, it suggests that assessing the current personality functioning of individuals with AUD has beneficial effects on treatment planning. Because the responsibility domain predicts drinking levels at treatment end, clinicians would benefit from knowing which individuals score low on this domain. The association between being responsible and drinking at low-risk levels following treatment is not surprising in itself, but has notable implications from the perspective of personalised medicine. Individuals who are less responsible will probably need more personalised treatment to obtain favourable outcomes. In particular, these individuals may require additional support to complete treatment, and care should be taken to ensure they attend the planned sessions. Second, concerning the therapeutic alliance, it may be necessary to approach individuals with lower levels of responsibility with particular attention, because they also might attend therapy but struggle to commit to therapeutic tasks and goals.

The present study has several limitations. First, we examined alcohol use only at treatment end, which is a short-term outcome. Short follow-up periods are a problem in most studies on AUD treatment outcomes, and future studies should be designed with more extended follow-up periods. Second, our sample is relatively small, possibly affecting the power of the study to detect effects. Relatedly, due to limitations in the data, we had to dichotomise the outcome variable, which necessarily led to a loss of information and statistical power. Third, because the sample mainly consisted of persons who had completed the treatment. the results are therefore probably generalisable only to groups completing treatment. We know from a study of the same sample that those discontinuing treatment had lower executive functioning than those completing treatment, and the latter group constitutes the sample of the current study.^21^ Other factors may affect individuals who discontinue treatment. In contrast, neuropsychological functioning did not predict drinking outcomes in the present study. Consequently, different factors and paths may predict discontinuation compared with those predicting favourable outcomes upon completing treatment. Fourth, as the majotity of of the sample were out-patients, the results may apply to persons in the higher-level functioning of the AUD spectrum. Fifth, as a study on routine practice, we had limited access to outcome measures other than AUDIT. Using biological markers such as PEth or the Timeline Follow-Back method might have given different and more nuanced results. In addition, aside from attendance, the study used baseline variables as predictors of outcome; more frequent monitoring of outcomes throughout treatment could have offered more detailed information on change trajectories. Future studies with larger samples could investigate these questions, using, for example, latent profile analyses to provide a more nuanced picture of trajectories of change following treatment.

While attendance seems to influence high-/low-risk drinking at the end of treatment, the personality domain of responsibility appears to have a more substantial impact. Strategies to improve attendance, particularly among individuals with low levels of responsibility, would be valuable in enhancing their otherwise poorer prognosis. Clinicians are advised to screen for personality functioning before treatment when planning treatment for those with AUD.

## Data Availability

The data that support the findings of this study are not publicly available due to confidentiality issues. Please contact the corresponding author with any questions.

## Trial registration

Clinical Trials ID: NCT04822987, registered 26 March 2021 (https://www.clinicaltrials.gov/study/NCT04822987?id=NCT04822987).
