# Predictors of high- and low-risk drinking after group treatment for alcohol use disorder – CORRIGENDUM

**DOI:** 10.1192/bjo.2026.10992

**Published:** 2026-02-10

**Authors:** Kristoffer Høiland, Espen Kristian Ajo Arnevik, Lien My Diep, Tove Mathisen, Anette Søgaard Nielsen, Jens Egeland

Since this article’s original publication, the authors have noticed that table 2 is mislabelled. This applies only to the variables, and the actual values in the table are correct.

The corrected table is below.

**Table 2:** Associations between key variables pretreatment and outcome at treatment end (low- versus high-risk drinking)

Key variables	Total *N* (92)	Low-risk 39 (42%)	High-risk 53 (58%)	*P*-value	Missing
Demographics					
Male/female, *n*	61/31	26/13	35/18	0.950	0
Age (M/SD)	50.3 (11.2)	50.0 (9.8)	50.5 (12.2)	0.820	0
Education (years) (M/SD)	13.1 (2.5)	12.8 (2.4)	13.3 (2.6)	0.391	3
Higher education, *n* (%)	36 (40.0)	15 (39.5)	21 (40.4)	0.931	2
Married, *n* (%)	28 (30.4)	14 (35.9)	14 (26.4)	0.329	0
Previous SUD treatment, median (IQR)	1.0 (0–2.5)	1.0 (0–2)	1.0 (0–3.5)	0.251	4
Substance use					
AUDIT, median (IQR)	22.0 (19.0–27.0)	21.5 (14.7–22.6)	23.0 (21.4–25.1)	0.138	3
DUDIT, median (IQR)	0 (0–12.0)	0 (1.9–8.1)	0 (4.1–10.2)	0.154	3
Percentage attendance treatment, median (IQR)	84.4 (68.4–94.9)	93.0 (82.5–92.6)	76.5 (72.8–81.7)	**0.002** ^a^	0
Cognitive functioning					
General Ability Index (GAI) (M/SD)	95.0 (12.8)	95.2 (13.8)	94.9 (12.2)	0.904	2
Working Memory (M/SD)	8.9 (2.2)	8.6 (2.3)	9.2 (2.1)	0.183	2
Executive functioning domain (M/SD)	9.4 (2.4)	9.6 (2.7)	9.3 (2.2)	0.642	3
Mental Speed Domain (M/SD)	9.0 (2.3)	8.8 (2.5)	9.1 (2.2)	0.574	3
Mental health and personality					
SCL-90-R (GSI) (M/SD)	0.98 (0.59)	0.93 (0.59)	1.0 (0.59)	0.623	1
Self-Control (M/SD)	5.33 (0.96)	5.61 (0.74)	5.13 (1.06)	0.**024**	9
Identity Integration (M/SD)	4.11 (0.85)	4.29 (0.77)	3.98 (0.89)	0.099	9
Responsibility (M/SD)	4.75 (0.78)	5.14 (0.65)	4.47 (0.75)	**<0.001** ^a^	9
Relational Capacities (M/SD)	4.57 (0.76)	4.76 (0.73)	4.44 (0.76)	0.056	9
Social Concordance (M/SD)	6.30 (0.69)	6.51 (0.66)	6.16 (0.69)	**0.021**	9
Quality of life					
Physical (M/SD)	13.12 (2.73)	13.27 (2.66)	13.01 (2.80)	0.674	6
Psychological (M/SD)	11.94 (2.69)	12.12 (2.52)	11.80 (2.84)	0.589	6
Social (M/SD)	13.05 (2.83)	13.19 (2.93)	12.95 (2.79)	0.704	6
Environmental (M/SD)	14.49 (2.46)	14.65 (2.70)	14.36 (2.28)	0.583	6

All predictor variables were examined at baseline, except for the percentage of treatment attendance. Group differences are described based on two-tailed *t*-tests, means and standard deviations, or based on Mann–Whitney *U*-tests, medians and IQR for non-normal variables and chi-square tests for categorical variables.

Mental health: GSI from SCL90-R: personality domain scores from Severity Indices of Personality Functioning; quality of life: domain scores from World Health Organization Quality of Life Scale.

SUD, substance use disorder; IQR, interquartile range; AUDIT, Alcohol Use Disorders Identification Test; DUDIT, Drug Use Disorders Identification Test; SCL-90-R (GSI), Symptom Checklist 90 (General Symptom Index).

a. Significant following Bonferroni correction.

*P*-values <0.05 are indicated in bold.
